# Scaffold Stiffness Influences Cell Behavior: Opportunities for Skeletal Tissue Engineering

**DOI:** 10.2174/1874325000802010103

**Published:** 2008-05-29

**Authors:** Roel G.M Breuls, Timothy U Jiya, Theo H Smit

**Affiliations:** 1 Department of Physics and Medical Technology, VU University Medical Center, Research Institute MOVE, Amsterdam, The Netherlands; 2 Department of Orthopedics, VU University Medical Center, Amsterdam, The Netherlands; 3 Sketetal Tissue Engineering Group Amsterdam (STEGA), The Netherlands

**Keywords:** Orthopaedics, tissue engineering, scaffold, substrate stiffness, cell mechanics, bone, cartilage, tendon, mechanotransduction.

## Abstract

Skeletal defects resulting from trauma, tumors, or abnormal development frequently require surgical treatment to restore normal tissue function. To overcome the limitations associated with conventional surgical treatments, several tissue engineering approaches have been developed. In particular, the use of scaffolds enriched with stem cells appears to be a very promising strategy. A crucial issue in this approach is how to control stem cell behavior. In this respect, the effects of growth factors, scaffold surface characteristics, and external ‘active’ loading conditions on stem cell behavior have been investigated. Recently, it has become clear that the stiffness of a scaffold is a highly potent regulator of stem cell differentiation. In addition, the stiffness of a scaffold affects cell migration, which is important for the infiltration of host tissue cells. This review summarizes current knowledge on the role of the scaffold stiffness in the regulation of cell behavior. Furthermore, we discuss how this knowledge can be incorporated in scaffold design which may provide new opportunities in the context of orthopedic tissue engineering.

## INTRODUCTION

Skeletal defects as a result of injury or disease form a significant problem in health care with major socioeconomic impacts. Considering bone defects, traditional treatment modalities often use autograft and allograft cancellous bone. While these surgical treatments have increased the quality of life for many patients they have some important limitations. The use of autografts may be limited by scarceness of donor tissue and may cause donor site morbidity associated with infection, pain, and hematoma [1-6]. In addition, harvesting autografts requires a second surgical procedure, potentially increasing the risk of complications and cost of treatment. Allograft bone introduces the risk of host rejection and infection [7-11] and may also suffer from a limited supply. Restoration of cartilage and ligaments with autografts and allografts largely suffer from the same limitations.

Tissue engineering has been heralded as an alternative treatment strategy that may circumvent the problems associated with autograft and allograft procedures. Early tissue engineering approaches have used a variety of a-cellular scaffolds, including synthetic scaffold materials fabricated from hydroxyapatite, tricalcium phosphate ceramics, metal alloys, and various polymers such as polylactic acid (PLA) and polyglycolic acid (PGA) [12-15]. Furthermore, natural scaffolds consisting of extracted extracellular matrix (ECM) proteins such as collagen and fibrin have been used for tissue engineering (reviewed in [16]). The function of these acellular scaffold materials is to provide mechanical stability at the defect site, to stimulate the ingrowth of cells from healthy residual tissues *in situ*, and to guide the complex process of tissue formation.

A new generation of tissue engineering approaches incorporates the use of cells, which are seeded onto a scaffold to enhance the efficacy of tissue regeneration [17, 18]. The basic idea in this approach is to harvest cells from a patient, to seed these cells onto a scaffold material, and to subsequently implant the cell-seeded scaffold into the patient. The cells should produce the new tissue while the scaffold material gradually disintegrates, leaving no harmful traces in the body.

**Fig.(1):**
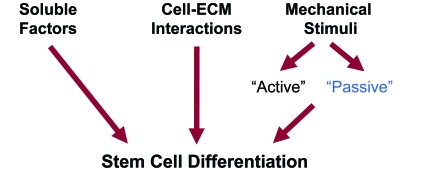
Stimuli affecting stem cell differentiation. Stem cell differentiation is affected by soluble factors, biochemical cell-matrix interactions, and mechanical stimuli. Mechanical stimuli can be subdivided in ‘active’ and ‘passive’. The ‘active’ forces are external mechanical forces acting on a scaffold during normal activity. The stiffness of a scaffold is a key ‘passive’ mechanical cue that affects stem cell differentiation.

Stem cells appear to be one of the most promising cell types to use in engineering applications. A central issue in the utilization of stem cells is how to pursue the stem cells to differentiate into the desired lineage and to restore tissue function. Unfortunately, there is not a simple answer to this question since stem cell differentiation is a highly complex and multi-factorial process. *In vivo*, stem cells are subjected to a complex array of biophysical and biochemical signals. These signals are processed and ‘integrated’ by the stem cells to regulate stem cell differentiation (Fig. **1**).

It has long been appreciated that soluble biochemicals such as growth factors, cytokines, and chemokines affect the morphogenesis of skeletal tissues. For example, growth factors from the TGFb superfamily notably the bone morphogenetic proteins (BMPs) are highly potent stimulators for skeletal tissue formation (reviewed in [19-24]). Furthermore, ECM proteins such as collagens, glycosaminoglycans, and proteoglycans provide instructive messages to cells that are transmitted across the cell membrane *via *the transmembrane receptors that recognize these ECM proteins. These cell-ECM interactions influence cell behavior either directly or through crosstalk with growth factors [25-29]. Furthermore, stem cells have been shown to respond to external mechanical loading (reviewed in [30-32]). For skeletal tissues, these ‘active’ external mechanical forces are required to maintain normal cell function [33-35]. Physical properties of a scaffold can also influence cell behavior. Examples include surface characteristics such as roughness [36-38], micro- and nano-topography [39, 40], surface energy [37], and porosity [41-44].

Only recently, it has been appreciated that also the stiffness of the ECM is a highly potent regulator of stem cell behavior [45-47]. The stiffness of the ECM acts as a ‘passive’ mechanical cue that tends to be more selective than soluble factors [47]. This review briefly describes how cells sense the stiffness of a scaffold and discusses the role of the scaffold stiffness in regulating cell behavior. In addition, the influence of scaffold stiffness on cell behavior in combination with biochemical stimuli is considered. Finally, scaffold stiffness is discussed as an opportunity to control cell behavior in the context of skeletal tissue engineering.

## SUBSTRATE STIFFNESS

Cells receive mechanical feedback from the substrate to which they adhere, even in the absence of externally applied forces. Here, ‘substrate’ refers to any material to which cells adhere, for example the ECM, a scaffold, or a culture flask. In recent years, researchers have been extensively investigating the role of the stiffness of a substrate as one of the key parameters that affects cell behavior. A key motivation for these studies was that cells *in vivo* often encounter a relatively soft environment, whereas conventional tissue culture flasks are very rigid. This led to the development of 2D *in vitro* model systems which use polymer gel substrates with tunable elastic properties that are coated with specific ECM proteins for cell attachment. In particular, polyacrylamide gels have been widely used because these gels can be tuned within a wide range of stiffness that mimic those of *in vivo* tissues (reviewed in [46, 48-51]). An essential characteristic of these *in vitro* ECM mimics is that the effects of substrate stiffness and substrate biochemistry on cell behavior can be independently studied. Commonly chosen stiffness values for the PA gels are in the range of ~0.5 kPa (brain tissue), ~10 kPa (muscle tissue), and >30 kPa (pre-mineralized bone) [46]. These gels are often referred to as ‘soft’, ‘intermediate stiff’, and ‘stiff’.

### Cell Probing of Substrate Stiffness

**Fig.(2):**
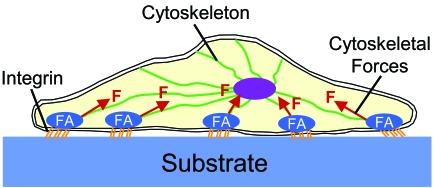
Schematic representation of a cell which has attached to a substrate. Cells attach to a substrate with transmembrane molecules called integrins. When cells bind to a substrate, integrins begin to cluster which leads to the formation of focal adhesions (FA). The maturation of focal adhesions requires the application of mechanical forces (F) to these adhesions which can be generated by the cytoskeleton.

Mechanotransduction is the process by which cells convert mechanical stimuli into a chemical response [52]. This plays an essential role in the probing of matrix stiffness and is currently subject of extensive research. It is beyond the scope of this review to provide a detailed description of the highly complex signaling cascades involved in mechanotransduction. Instead, we focus on a basic understanding of how cells probe the stiffness of a substrate. For a more detailed explanation we refer the reader to recent reviews on this topic [45, 53-57]. Cells bind to a substrate with transmembrane molecules called integrins, which have an extracellular domain that attaches to the substrate and an intracellular domain that connects to the cytoskeleton (Fig. **2**). When cells bind to the ECM, integrins begin to cluster, which leads to the recruitment of structural and signaling proteins to form so-called focal adhesions at the site of integrin clustering. The formation and maturation of focal adhesions requires the application of mechanical forces to these adhesions. Cells can actively generate these forces themselves using actin-myosin complexes which are part of their cytoskeleton. This is where substrate stiffness comes into play. On a hard substrate, cells generate large forces which leads to the formation of mature focal adhesions and a highly organized cytoskeleton with abundant stress fibers [45, 58]. In contrast, a soft substrate cannot provide enough resistance to counterbalance large, cell-generated forces. Therefore, on soft substrates cells do not develop abundant stress fibers and generate smaller forces. In other words, a cell responds to differences in substrate stiffness and adjusts its ‘musculoskeletal system’ (i.e. the cytoskeleton) appropriately.

Changes in cytoskeletal organization are important, because the cytoskeleton is involved in many signaling pathways that transfer mechanical feedback into chemical responses. Furthermore, the cytoskeleton also determines the shape of a cell, which in turn is intimately connected to cell behavior (reviewed in [56, 59]). This was clearly demonstrated by cell biophysicist Christopher Chen and co-workers who showed that cell morphology can regulate differentiation of stem cells [60]. This group cultured stem cells on adhesive islands with different sizes to force stem cells to adopt a particular cell shape. On small islands, cell spreading was impaired due to space limitations, whereas cells normally spread on larger islands. In culture medium containing both adipogenic and osteogenic differentiation factors, it was found that cells became adipocytes on the small islands but committed to the osteogenic lineage on the large islands [60]. In other words, when these cells received culture medium supporting both osteogenic and adipogenic differentiation, the cell shape determined their fate. Even more intriguing, when cells were cultured on large islands in adipogenic medium, the cells still became osteogenic; vice versa, cells on small islands in osteogenic medium still became adipogenic. Thus, cell shape could drive stem cell differentiation and overrule the instructive messages provided by soluble differentiation factors. The cytoskeleton, in the form of actin-myosin generated tension proved to be essential in the stem cell commitment process.

### Substrate Stiffness and Cell Behavior

**Fig.(3):**
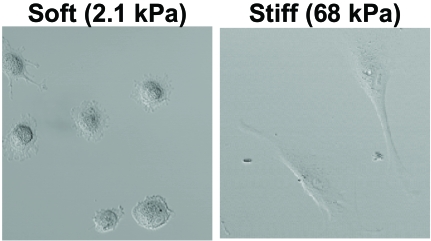
Fibroblasts cultured in our lab on ‘soft’ and  ‘stiff’ polyacrylamide gels coated with collagen I, four hours after plating. On a soft gel, cells adopted a round morphology whereas these cells spread on stiff gels.

Since substrate stiffness influences the cytoskeletal organization, it also affects cell morphology. Various studies indicated that stiffer substrates generally promote cell spreading, whereas soft substrates induce a more rounded cell shape (Fig. **3** and refs. [58,61,62]). From the above, it is not surprising that these changes in cell morphology are accompanied by changes in cell behavior, including cell differentiation. Discher and co-workers showed that muscle precursor cells optimally differentiate into myotubes on substrates with a stiffness that mimics that of muscle tissue [63, 64]. In addition, this group demonstrated that substrate stiffness can control the differentiation of stem cells [47]. Naïve mesenchymal stem cells were shown to specify lineage and commit to phenotypes with extreme sensitivity to substrate stiffness. Soft gels that mimic brain tissue are neurogenic, stiffer matrices that mimic muscle tissue are myogenic, and rigid gels prove osteogenic. During the initial week of culturing, reprogramming of these lineages is possible with the addition of soluable induction factors, but after several weeks in culture, the cells commit to the lineage specified by matrix stiffness [47].

Apart from changes in cell differentiation, the substrate stiffness also affects cell migration. This is important for the ingrowth of cells from host tissues, for example for the vascularization of a scaffold. It was reported that normal rat kidney epithelial cells and fibroblasts migrate faster on softer substrates compared to stiff substrates [49], whereas vascular smooth muscle cells showed maximal migration on intermediate stiff substrates [65]. It was demonstrated that these effects are mediated by changes in focal adhesion formation and myosin-generated forces [49]. Furthermore, when fibroblasts were cultured on a substrate with a soft and a stiff side, they preferred migrating into the direction of the stiff side (a phenomenon called ‘durotaxis’) [66]. Considering tissue formation, it was shown that fibroblasts merge to form tissue-like structures on soft substrates whereas these cells migrated away from one another on stiff substrates [67]. Similar behavior was observed for epithelial cell lines and explants from neonatal rat hearts. The driving force behind this merging of cells on soft substrates is a combination of weak adhesions and myosin II contraction [67].

Substrate stiffness also influences cell growth and viability. For example, osteoblasts showed increased proliferation, motility, and deposition of mineral on hard surfaces compared to soft substrates [68]. Importantly, the responses to substrate stiffness are different for normal and oncogenic cells. Non-transformed fibroblasts showed a decrease in the rate of DNA synthesis and an increase in the rate of apoptosis on soft substrates [69]. In contrast, transformed cells maintained their growth and apoptosis characteristics regardless of substrate rigidity. This indicates that feedback from substrate stiffness is required for proper regulation of cell growth and survival.

In the polyacrylamide gel system, cells are attached to a substrate as depicted in Fig. (**2**). This means that the cell only attaches to a substrate on one side, thus experiencing a two-dimensional (2D) situation. Recently, the effect of substrate stiffness on cell behavior was studied in three-dimensional (3D) model systems [70-72], whereby cells are embedded in a gel consisting of ECM proteins. These 3D *in vitro* model systems provide a better representation of the 3D extracellular environment that cell encounter *in vivo* [59, 73]. A drawback, however, is that it is difficult to independently control substrate stiffness and ECM biochemistry in these 3D systems. It appeared that matrix stiffness plays an important role in modifying cell behavior also in a 3D environment. For example, smooth muscle cell morphology was much rounder in stiff compared to soft 3D ECM analogs [71]. Interestingly, there can be important differences in cellular responses between 2D and 3D culture models. For example, the migration of human prostate cancer cells in 2D and 3D were shown to exhibit diametrically opposite behaviors [74].

In summary, current data demonstrate that substrate stiffness affects essential cell behavior including differentiation, migration, and growth. The ‘musculoskeletal system’ of the cell, i.e. the cytoskeleton, plays a crucial role in translating feedback from the substrate stiffness into cell behavior.

### Substrate Stiffness and Biochemical Factors

Only few studies investigated the combined effects of biochemical and biophysical factors on cell behavior. As already mentioned, cell shape and substrate stiffness can supersede biochemical signaling under certain conditions [47, 60]. In addition, soluble induction factors appear to be less selective than matrix stiffness in driving stem cell differentiation, and cannot reprogram stem cells that were precommitted for weeks on a matrix with a certain stiffness [47]. On the other hand, a ‘correct’ substrate stiffness and soluble induction factors can combine to induce a more complete cell differentiation [47]. Considering substrate stiffness and ligand density (i.e. the availability of attachment sites), it was found that smooth muscle cells spread maximally on intermediate ligand densities on stiff substrates [62]. However, on soft substrates these cells were relatively insensitive to ligand density and showed a more rounded morphology [62]. Stimuli induced by specific ECM proteins also appear to be dependent on the stiffness of a substrate. It was found that collagen V, an ECM protein that is highly expressed during tissue development and repair, modifies cell morphology and migration on substrates with a tissue-like stiffness, but not on hard substrates (R.G.M. Breuls, submitted).

## OPPORTUNITIES FOR SKELETAL TISSUE ENGINEERING

The ECM integrates many functions including the provision of structural support, attachment sites for cell surface receptors, and a reservoir for signaling molecules [75]. For a successful tissue engineering approach the complex tasks of the ECM need to be taken over by a scaffold material, which outlines the challenges for a scaffold designer. While it is virtually impossible to mimic the full complexity of the natural ECM, one may attempt to incorporate the most essential features in a scaffold. The stiffness of a scaffold appears to be one of these key variables.

### Mechanical Properties of a Scaffold

When focusing on the mechanical properties, a scaffold should be strong enough to withstand the loads that act on a skeletal tissue at the defect site. Furthermore, the stiffness is important because it affects the strains acting on a cell while being attached to a scaffold [76]. For example, an extremely stiff titanium scaffold will clearly not transfer external loads to cells because it will not deform under physiologic loading conditions. However, the influence of mechanics on cell behavior is not limited to external loading. Even in the absence of external loads, the stiffness of a scaffold is important for the regulation of cell behavior.

Since substrate stiffness affects many different processes, such as cell growth, migration, and differentiation, it is difficult to provide a general guideline for a suitable scaffold stiffness that optimally stimulates tissue regeneration. Nevertheless, to optimally promote stem cell differentiation, current knowledge suggests that the scaffold stiffness should match the *in vivo* stiffness of the skeletal tissue under consideration. It is important to emphasize that a scaffold should probably exhibit the stiffness of a *developing* skeletal tissue, which might be lower than the stiffness of a mature tissue. To promote the invasion of the scaffold with cells from surrounding host tissue, a stiffer scaffold might be more favorable [66]. This may set conflicting requirements for the scaffold stiffness with respect to stem cell differentiation and invasion of host cells.

### Scaffold Surface Biochemistry

Since substrate stiffness and biochemical stimuli interact to determine cell behavior, both parameters need to be controlled in scaffold design. Natural polymers such as collagen provide ligands for cell binding, however, the mechanical properties of these natural polymers are difficult to control. Synthetic polymers on the other hand generally offer the advantage of better control over the material properties but may require additional treatment to incorporate specific cell binding sites. By determination of the specific amino acid sequences of natural proteins that cells bind to, researchers were able to mimic cell binding sites in custom made peptide sequences that can be incorporated in synthetic scaffolds [77, 78]. Therefore, latest trends in scaffold design have been focusing on incorporating spatially well-defined cell binding sites in synthetic materials [79-82]. Such synthetic mimics of natural ECM materials have already been successfully applied for the regeneration of bone defects in rats [83, 84].

### Utilization of Scaffold Stiffness in Tissue Engineering Applications

Most research on the use of stem cells in skeletal tissue engineering applications have relied on bone marrow derived stem cells. The use of stem cells extracted from bone marrow may require culturing of the stem cells *in vitro* in order to enrich and expand the stem cell population. Such procedures are costly and quality insurance in the clinical setting may be difficult to achieve. Cell culturing on conventional hard tissue culturing flasks might introduce another problem, however, since these hard materials can induce an undesired preconditioning of the stem cells as a consequence of an inappropriate stiffness [47]. Therefore, it might be useful to consider expansion of cells on substrates with a stiffness that compares to the stiffness of native tissue. However, cells may not proliferate on soft substrates that mimic native tissue. The problem is that cells either proliferate or synthesize matrix and differentiate at high rates. Stiff substrates seem to enhance proliferation, whereas soft substrates are more dedicated to differentiation.

The introduction of alternative sources for stem cells such as the adipose-derived stem cells may allow for a different approach that solves this problem [85, 86]. Adipose-derived stem cells can be obtained in relative large quantities, which circumvents the need for cell culturing [87-89]. This has led to the formulation of a so-called one-step surgical procedure that avoids *in vitro* cell culturing [90]. In this procedure, stem cells are harvested form the patient, immediately seeded onto a scaffold, and implanted into the patient, all in a single surgical procedure. In this approach, an appropriate scaffold may circumvent undesired pre-conditioning of stem cells and, ideally, provide biochemical and mechanical stimuli that support the process of stem cell differentiation.

Generally speaking, most synthetic polymers that are currently used in orthopedic practice are stiff materials with elastic moduli in the range of ~3 to 2000 MPa [91]. This suggests that these polymers could be suboptimal to stimulate stem cell differentiation, in particular for softer tissues. The latest trend, however, is that polymers such as PLA are used to create loosely packed fibrous meshes that have a much lower stiffness [92-98]. As a consequence, the cells ‘feel’ a relative low stiffness when they are embedded in these fibrous meshes. This opens up exciting new opportunities with these FDA-approved biodegradable materials. The tissue engineer may fabricate a scaffold with a stiffness that is suitable to promote the desired cell behavior. This might be the stimulation of cell proliferation, differentiation, or even the migration of host cells, depending on the specific requirements *in situ*. Obviously, a vast amount of research is needed to further investigate the effects of substrate stiffness in combination with other variables such as pore size in the context of these fibrous meshes.

## CLOSING REMARKS

Recently, the proof of concept of stem cell based approaches in orthopaedics has been demonstrated in animal models. However, at present, only very few studies on humans exists [99-101], which had only limited success [17]. Possibly, a better understanding of the complex multi-factorial process regulating stem cell behavior may lead to the often anticipated breakthrough in tissue engineering. This review focused on the importance of the stiffness of a scaffold in tissue engineering applications. Scaffold stiffness can guide stem cell differentiation and affects cell migration, the latter being important with respect to cell infiltration from host tissues. Although biochemical stimuli are clearly very important in the regulation of cell behavior, it has become clear that a scaffold with an inappropriate stiffness may frustrate the process of tissue regeneration. On the other hand, a scaffold with a well-chosen stiffness can stimulate the regeneration of new tissue and enhance the efficacy of biochemical stimuli. Thus, the scaffold stiffness may be an important variable to control stem cell behavior which provides new opportunities for tissue engineering.
